# Supplementary material for: Nse5/6 inhibits the Smc5/6 ATPase and modulates DNA substrate binding
Source: EMBO J. 2021 Jun 30;40(15):e107807. doi: 10.15252/embj.2021107807 (PMC8327961; doi:10.15252/embj.2021107807)
Supplement: Supplementary file 2 — Expanded View Figures PDF [file EMBJ-40-e107807-s008.pdf]

Expanded View Figures

Figure EV1. Structural views and conservation of Nse5 and Nse6.

A The Nse6 moiety of the Nse5/6 dimer structure is shown in front, back and side views in cartoon representation and in corresponding surface conservation displays (at the bottom left of each panel). The concave surface interacting with Nse5 shows highest residue conservation. Conservation colour code is indicated at the right.

B Front and back views of Nse5 in the Nse5/6 structure in cartoon and corresponding surface conservation representation. Display as in (A). Conservation colour code as in (A).

C Zoom view of contacts at the Nse5/6 interface formed by residues in helix α12 as well as the preceding loop in Nse5 with Nse6 helices α1 and α3.

D Verification of the Nse5/6 structure by BMOE cross-linking. Residues H368 in Nse6 and G56 in Nse5 (see structure with zoom-in on the left) were mutated to cysteines. Incubation with BMOE (30 s) of the mutant but not the wild-type complex led to robust cross-linking seen as a decrease in electrophoretic mobility of the cross-linked species in SDS–PAGE (right).

E (*left panel*) Superimposition of Nse6 helices α8-α11 with selected top hits from a DALI search in the Protein Data Bank in top and front view. Root mean square displacement (rmsd) values are given as indicator for the quality of the fit. *(right panel)* Similar analysis for the N-terminal region of Nse5 (helices α1- α6).

F Superimposition of the Nse5/6 crystal structure (this work; PDB: 7OGG) with a similar cryo-EM structure published while this work was in progress (PDB: 7LTO). The two independently derived models are highly similar (rmsd 0.829 Å).

Source data are available online for this figure.

 © 2021 The Authors

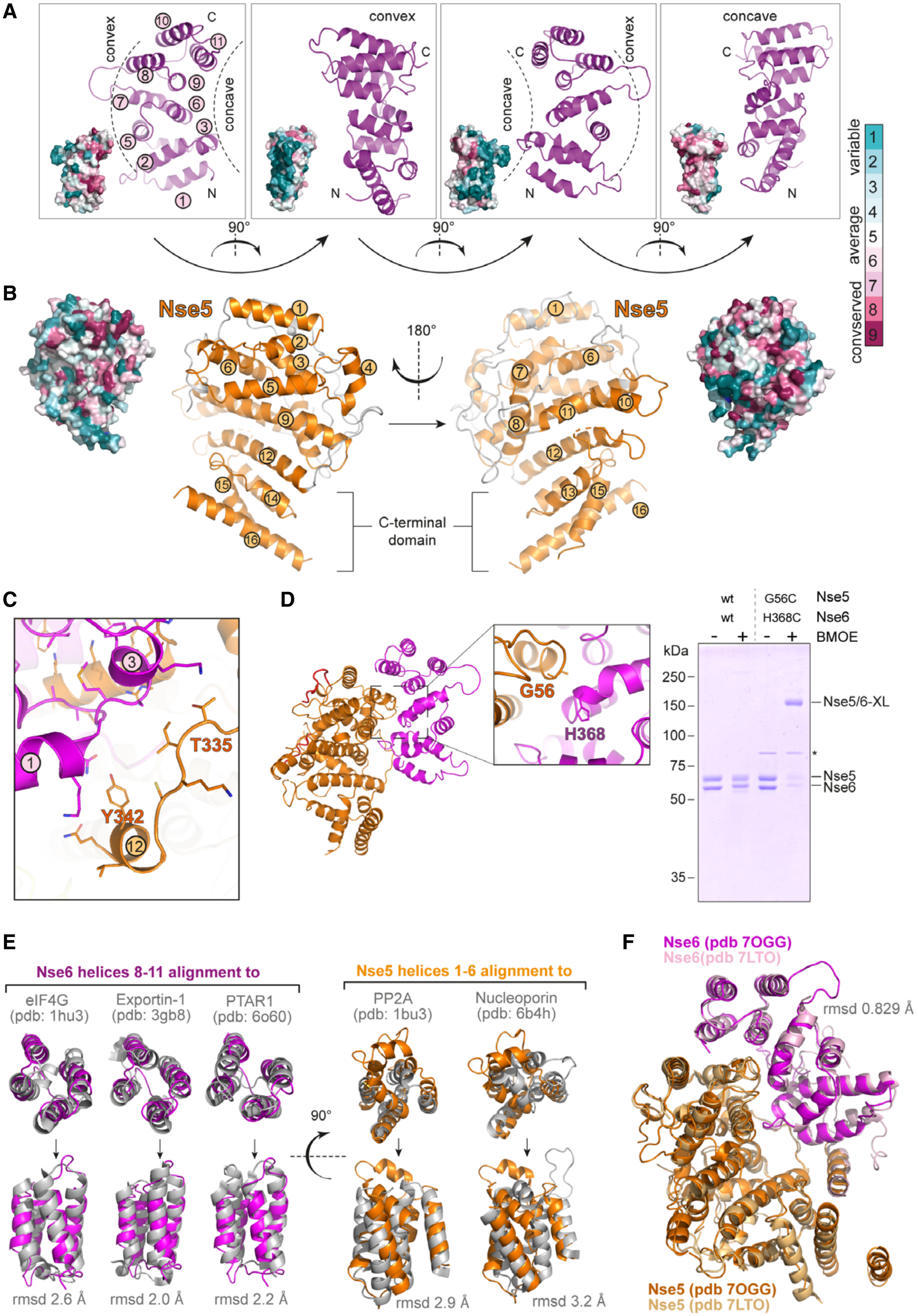
A
convex
concave
C
N
90°
variable
average
convserved
1
2
3
4
5
6
7
8
9
B
Nse5
180°
C-terminal domain
C
T335
Y342
D
wt
wt
G56C
H368C
Nse5
Nse6
BMOE
kDa
250
150
100
75
50
35
Nse5/6-XL
Nse5
Nse6
G56
H368
E
Nse6 helices 8-11 alignment to
eIF4G (pdb: 1hu3)
Exportin-1 (pdb: 3gb8)
PTAR1 (pdb: 6o60)
Nse5 helices 1-6 alignment to
PP2A (pdb: 1bu3)
Nucleoporin (pdb: 6b4h)
rmsd 2.6 Å
rmsd 2.0 Å
rmsd 2.2 Å
rmsd 2.9 Å
rmsd 3.2 Å
F
Nse6 (pdb 7OGG)
Nse6(pdb 7LTO)
rmsd 0.829 Å
Nse5 (pdb 7OGG)
Nse5 (pdb 7LTO)

Figure EV1.

Figure EV2. Smc5/6 interactions measured by pulldowns.

A Competition binding of a mixture of Nse5-His/Nse6 and Nse6(1-179)-CPD-His ("in") to immobilized Smc5/Smc6-Twin-Strep hexamer. Input ("in") and pulldown fractions were analysed by SDS–PAGE and Coomassie staining.

B Competition with excess Nse5/6 and Nse6 fragment. Smc5/6 hexamers were immobilized together with Nse5-His/Nse6 or Nse6(1–179)-CPD-His and washed with Nse6(1-179)-CPD-His or Nse5-His/Nse6, respectively. Input ("in"), pulldown and control pulldowns (without competition) were analysed by SDS–PAGE and Coomassie staining.

C Pulldown ("pd") assays examining the interaction of Smc5/Smc6-Twin-Strep ("TS") hexamer ("in") with either Nse6(1–179)-CPD-His or Nse6(86–179)-CPD-His. Fractions were analysed by SDS–PAGE and Coomassie staining showing that residues 1–85 in Nse6 are dispensable for interaction with the Smc5/6 hexamer.

D Spotting assay to determine sensitivity of yeast strains to UV irradiation and treatment with methyl methanesulphonate (MMS) or hydroxyurea (HU). A strain lacking the N-terminal region of Nse6 (*nse6(86-C)*) does not show increased sensitivity compared to the wild type, while a control strain (*smc6(R135E)*) recently described by others (Serrano *et al*, 2020) does.

Source data are available online for this figure.

 © 2021 The Authors

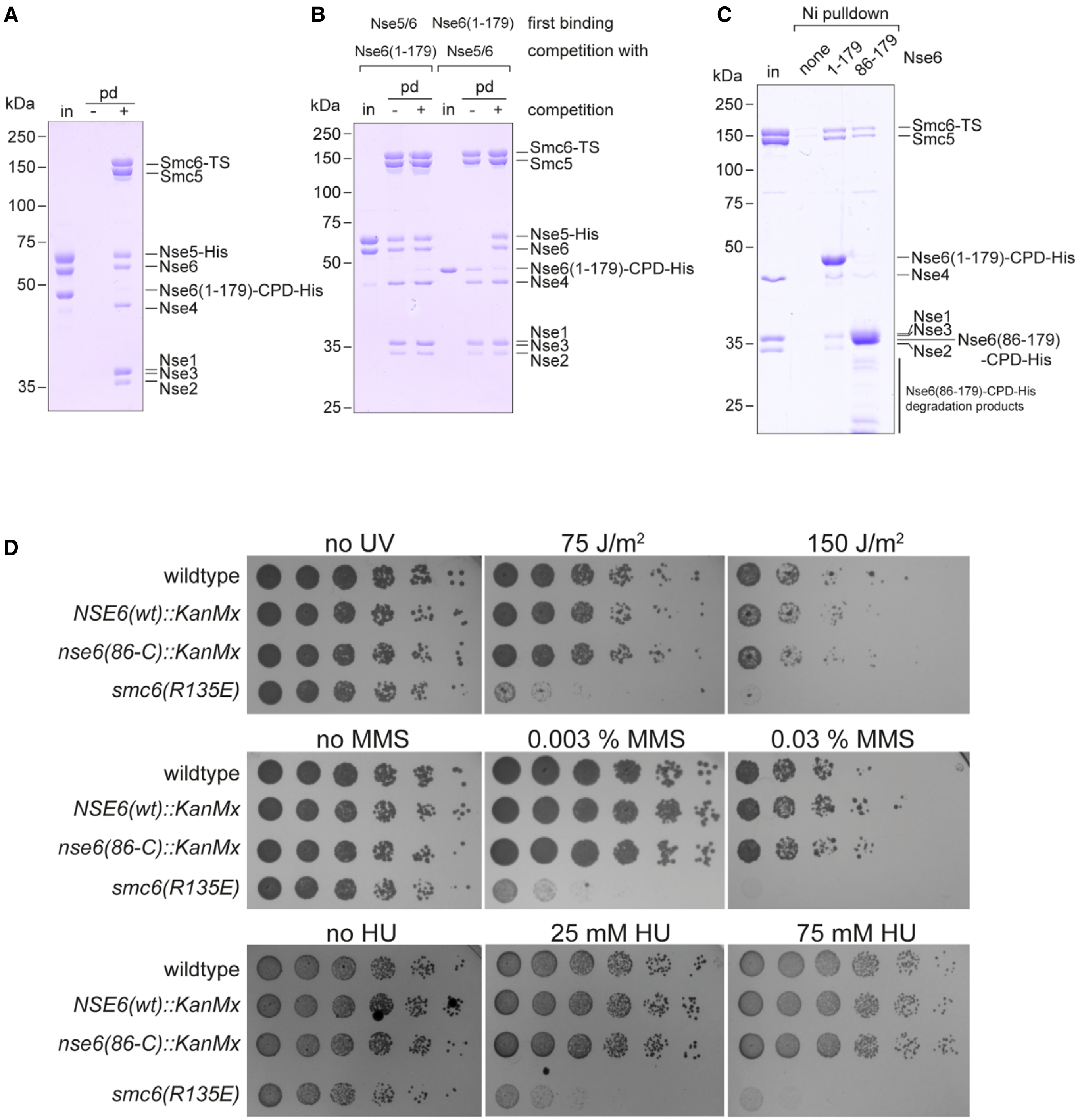
A
kDa
in
pd
-
+
250
150
100
75
50
35
Smc6-TS
Smc5
Nse5-His
Nse6
Nse6(1-179)-CPD-His
Nse4
Nse1
Nse3
Nse2
B
Nse5/6
Nse6(1-179)
first binding
competition with
competition
Smc6-TS
Smc5
Nse5-His
Nse6
Nse6(1-179)-CPD-His
Nse4
Nse1
Nse3
Nse2
C
Ni pulldown
none
1-179
86-179
Nse6
Smc6-TS
Smc5
Nse6(1-179)-CPD-His
Nse4
Nse1
Nse3
Nse2
Nse6(86-179)
-CPD-His
Nse6(86-179)-CPD-His
degradation products
D
no UV
75 J/m2
150 J/m2
wildtype
NSE6(wt)::KanMx
nse6(86-C)::KanMx
smc6(R135E)
no MMS
0.003 % MMS
0.03 % MMS
no HU
25 mM HU
75 mM HU

Figure EV2.

Figure EV3. ATP hydrolysis by Smc5/6 and mutants thereof.

A ATP hydrolysis rates for wild-type Smc5/6 hexamers and the Smc5(EQ)/Smc6(EQ) variant. Rates for the absence and presence of DNA substrates are given (per Smc complex per minute). Assays were performed in technical triplicates, and mean values are shown with error bars indicating standard deviations. Individual data points are also displayed.

B Cooperativity in ATP hydrolysis by the Smc5/6 hexamer. ATP hydrolysis rates were determined at different protein concentrations. Smc5/6 hexamers showed cooperative behaviour without DNA and with ^{40bp}DNA but not with ^{plasmid}DNA. Assays were performed in technical triplicates, and individual data points are displayed.

C Absence of cooperative in ATP hydrolysis by the Smc5/6 octamer. As in (B) using reconstituted octamers. Assays were performed in technical triplicates, and individual data points are displayed.

D Overview of values for ATP hydrolysis rates measured at an elevated ATP concentration (1 mM) (*left panel*) and fold-stimulation of ATP hydrolysis rates by addition of ^{40bp}DNA (conc. 1 μM), ^{plasmid}DNA (conc. 1.5 nM) in closed covalent circular (“circ”) or linearized form (“lin”) (*right panel*), or ^{40mer}ssDNA (conc. 1 μM). Assays were performed in technical triplicates, and mean values are shown with error bars indicating standard deviations. Individual data points are also displayed.

E Analysis of the plasmid used in ATPase assays on a 0.7% agarose gel stained with SybrSafe, either untreated or after treatment with Topoisomerase I. The plasmid is present as a mixture of supercoiled and relaxed form. Note that due to the large size the relaxed form does not properly enter the gel and is largely retained in the wells.

F Pulldown assay between hinge-less Smc5 and Smc6 sub-complexes carrying the “EQ” mutation that abolishes ATP hydrolysis (Smc5Δhinge(EQ)/Nse2/Nse4(C) and Smc6Δhinge(EQ)/Nse4N), respectively; see scheme on the left) in the presence or absence of ATP or ATPγS. The SDS–PAGE gel on the right shows that the complexes do not detectably interact in the absence of ATP but do so in its presence. Head engagement is clearly less efficient with the non-hydrolysable analogue ATPγS.

Source data are available online for this figure.

 © 2021 The Authors

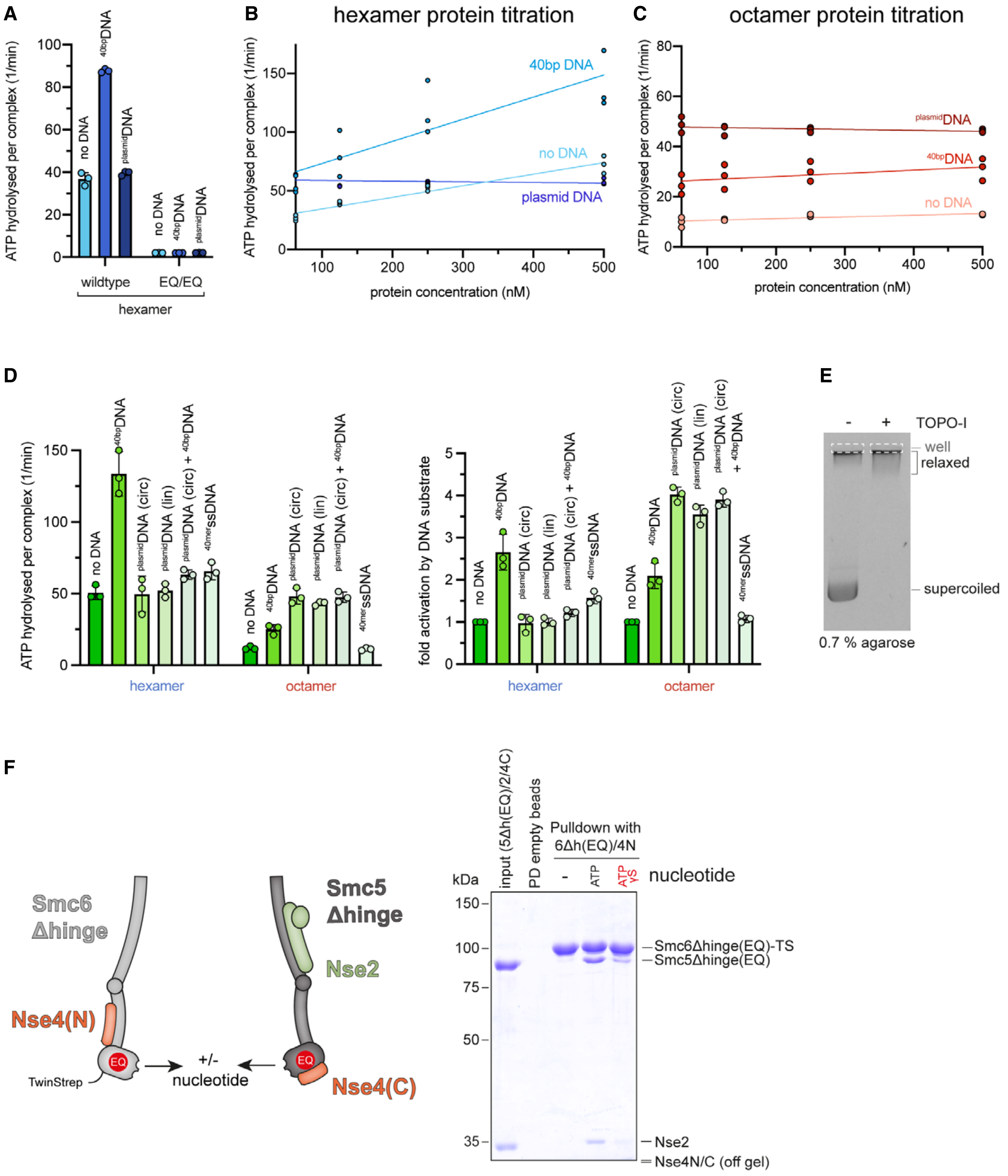
A
B
hexamer protein titration
C
octamer protein titration
ATP hydrolysed per complex (1/min)
no DNA
40bpDNA
plasmidDNA
wildtype
EQ/EQ
hexamer
40bp DNA
no DNA
plasmid DNA
protein concentration (nM)
plasmidDNA
40bpDNA
no DNA
D
no DNA
40bpDNA
plasmidDNA (circ)
plasmidDNA (lin)
plasmidDNA (circ) + 40bpDNA
40merssDNA
hexamer
octamer
fold activation by DNA substrate
E
TOPO-I
well
relaxed
supercoiled
0.7 % agarose
F
Smc6 Δhinge
Nse4(N)
TwinStrep
EQ
+/- nucleotide
Smc5 Δhinge
Nse2
Nse4(C)
input (5Δh(EQ)/2/4C)
PD empty beads
Pulldown with 6Δh(EQ)/4N
ATP
ATPγS
nucleotide
kDa
150
100
75
50
35
Smc6Δhinge(EQ)-TS
Smc5Δhinge(EQ)
Nse2
Nse4N/C (off gel)

Figure EV3.

Figure EV4. Design of reporter cysteines in Smc5/6.

A Hinge-Cys residues were chosen based on a homology model of the budding yeast Smc5/6 hinge domain built from the fission yeast hinge structure (Alt *et al*, 2017). Residues for cross-linking of the "south" interface (top; Smc5(N526C) and Smc6(N643C)) or the "north" interface (bottom; Smc5(V638C) and Smc6(N572C)) are indicated in green colours on the hinge structure in surface representation.

B Hinge-Cys cross-linking at the "north" hinge interface. Scheme indicates the location of engineered cysteines and their expected ability to cross-link in a rod-like and a ring-like conformation. High-molecular weight species were analysed by SDS–PAGE and Coomassie staining. Wild-type hexamer ("wt") is included as cross-linking control. Species occurring only in the presence of engineered cysteines are labelled by coloured arrowheads. Cross-linking efficiencies were calculated from the intensity of Coomassie-stained bands by comparing the band of the corresponding cross-linked species to the bands of unmodified Smc5 and Smc6. Numbers below the gel quantify the percentage of cross-linked protein species in the displayed gel.

C Strategy for the construction of models for the J-state and the E-state based on structural models of the archaeal Smc-ScpAB complex.

D Positions of J-Cys (in blue colours), CC-Cys (in purple colours) and E-Cys (in red colours) residues on models of the J-state and the E-state.

E E-Cys cross-linking in the context of an ATP hydrolysis-deficient (EQ) Smc5/6 complex. Rest as in EV4B.

Source data are available online for this figure.

 © 2021 The Authors

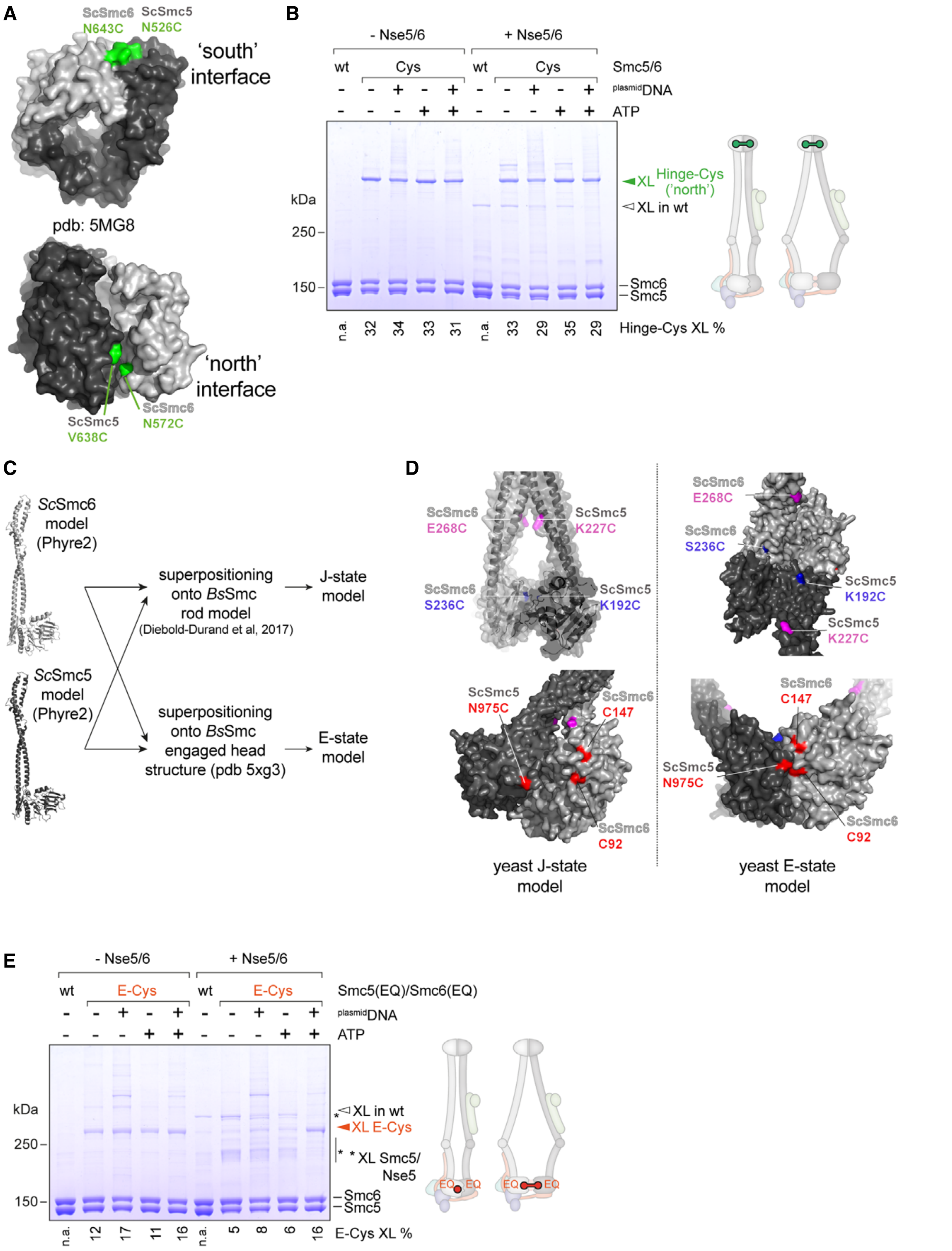
A
ScSmc6
N643C
ScSmc5
N526C
'south' interface
pdb: 5MG8
'north' interface
ScSmc6
N572C
ScSmc5
V638C
B
- Nse5/6
+ Nse5/6
wt
Cys
Smc5/6
plasmidDNA
ATP
kDa
250
150
XL Hinge-Cys ('north')
XL in wt
Smc6
Smc5
n.a. 32 34 33 31 n.a. 33 29 35 29
Hinge-Cys XL %
C
ScSmc6 model (Phyre2)
ScSmc5 model (Phyre2)
superpositioning onto BsSmc rod model
(Diebold-Durand et al, 2017)
J-state model
superpositioning onto BsSmc engaged head structure (pdb 5xg3)
E-state model
D
ScSmc6 E268C
ScSmc5 K227C
ScSmc6 S236C
ScSmc5 K192C
ScSmc5 N975C
ScSmc6 C147
ScSmc6 C92
yeast J-state model
yeast E-state model
E
- Nse5/6
+ Nse5/6
wt
E-Cys
Smc5(EQ)/Smc6(EQ)
plasmidDNA
ATP
kDa
250
150
XL in wt
XL E-Cys
XL Smc5/ Nse5
Smc6
Smc5
EQ
n.a. 12 17 11 16 n.a. 5 8 6 16
E-Cys XL %

Figure EV4.

Figure EV5. XL-MS of Smc5/6 octamers with and without substrates.

A Cross-links between Nse2 and Smc5/Smc6 proteins remain unaltered upon ^{plasmid}DNA addition.
B Changes in inter-subunit cross-links between Nse1/3/4 (left) and Nse5/6 (right) modules. Alternative representation of the same data shown in Fig 6B and C.
C Cross-links between Nse3 and Nse4 and between Nse3/Nse4 and Smc5/Smc6 detected in the Smc5(EQ)/Smc6(EQ) octamer without ATP and ^{plasmid}DNA (*left panel*) and with ATP and ^{plasmid}DNA (*right panel*).
D Same as in (C) for cross-links between Nse1/3/4/5/6 proteins.
E Pulldowns of the octameric complex ("in") via Smc6-Twin-Strep in the absence ("−") or presence ("+") of ATP and plasmid DNA under the same conditions as the ones used for XL-MS analysis. The Nse4/3/1 module is still present in the complex despite the severely reduced cross-linking efficiency.

Source data are available online for this figure.

 © 2021 The Authors

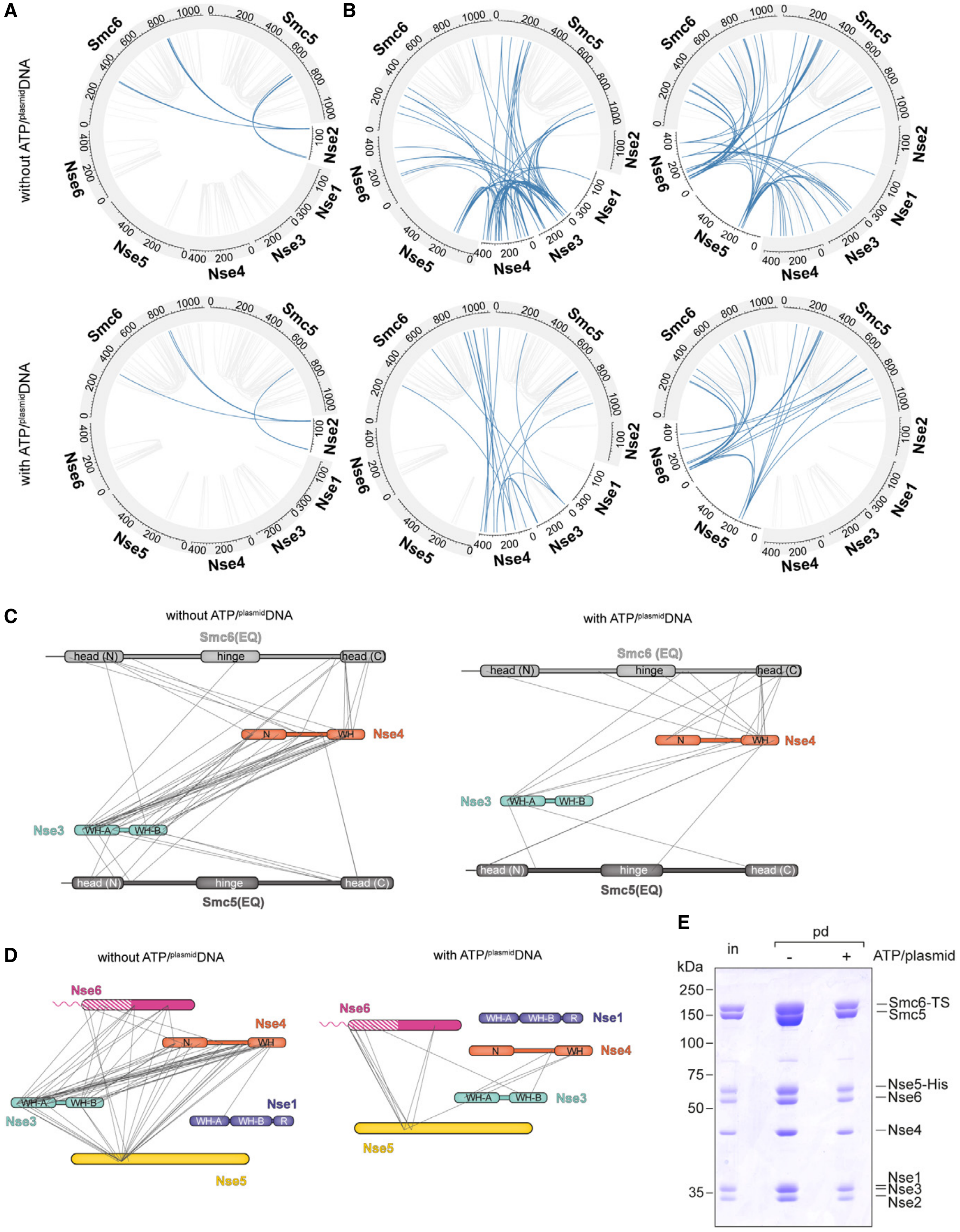
A
B
without ATP/plasmidDNA
with ATP/plasmidDNA
Smc6
Smc5
Nse2
Nse1
Nse3
Nse4
Nse5
Nse6
C
without ATP/plasmidDNA
with ATP/plasmidDNA
Smc6(EQ)
head (N)
hinge
head (C)
Nse4
N
WH
Nse3
WH-A
WH-B
Smc5(EQ)
D
Nse6
Nse1
R
Nse5
E
pd
in
-
+
ATP/plasmid
kDa
250
150
100
75
50
35
Smc6-TS
Smc5
Nse5-His
Nse6
Nse4
Nse1
Nse3
Nse2

Figure EV5.
