# Supplementary figures and images for: Nse5/6 inhibits the Smc5/6 ATPase and modulates DNA substrate binding
Source: EMBO J. 2021 Jun 30;40(15):e107807. doi: 10.15252/embj.2021107807 (PMC8327961; doi:10.15252/embj.2021107807)

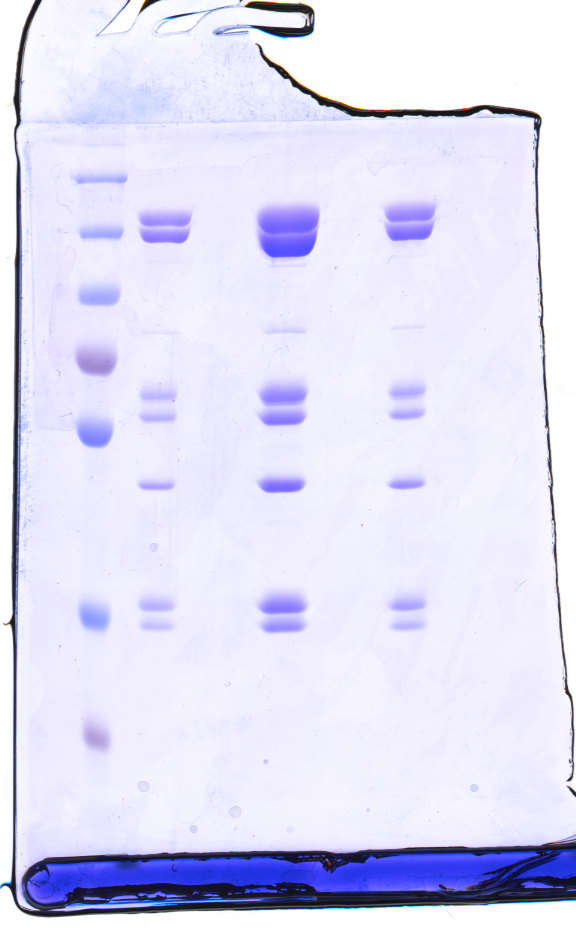

Supplement: Supplementary file 4 — Source Data for Expanded View [file EMBJ-40-e107807-s006.zip › Fig EV5E.tif]

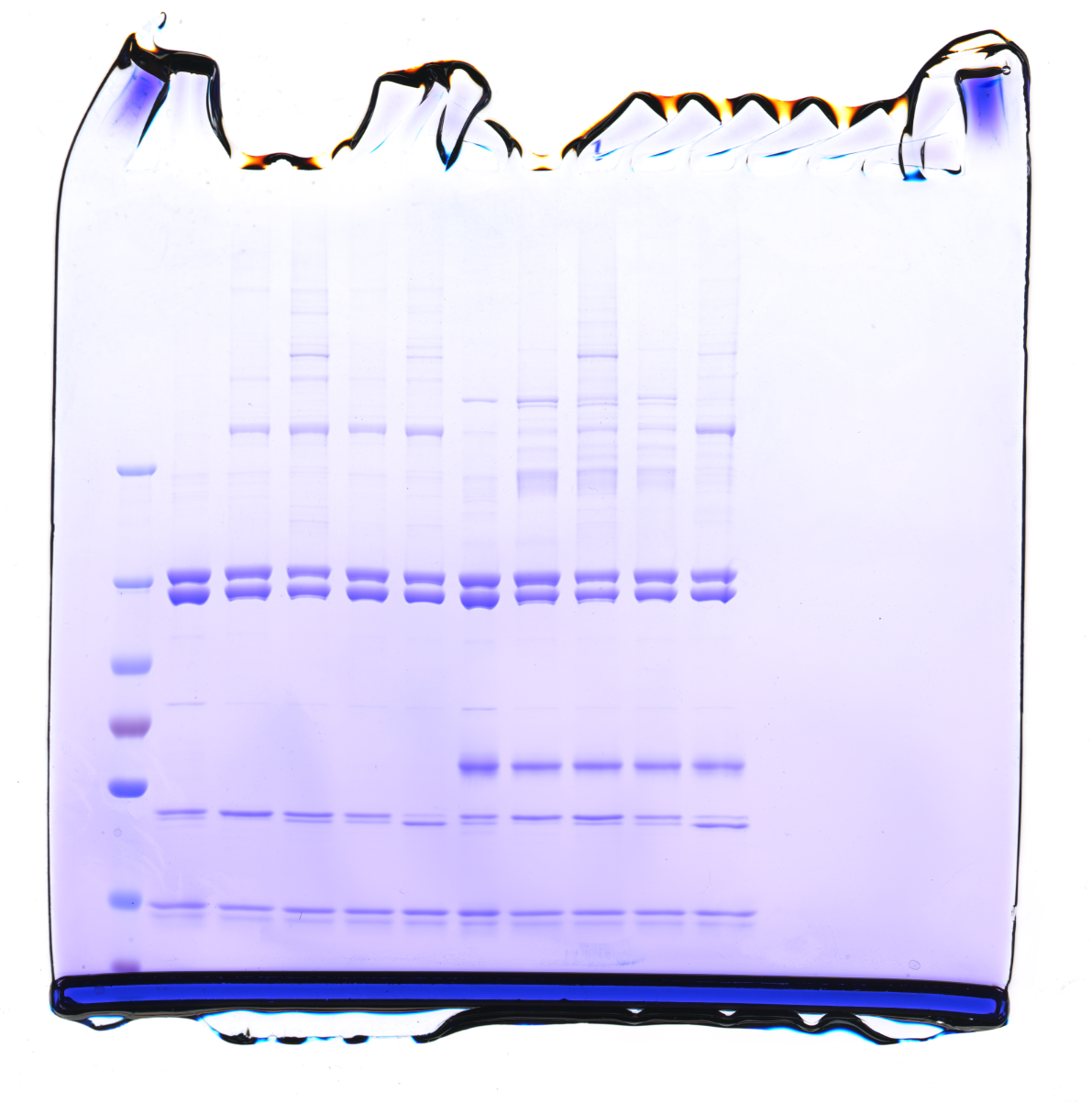

Supplement: Supplementary file 4 — Source Data for Expanded View [file EMBJ-40-e107807-s006.zip › Fig EV4E.tif]

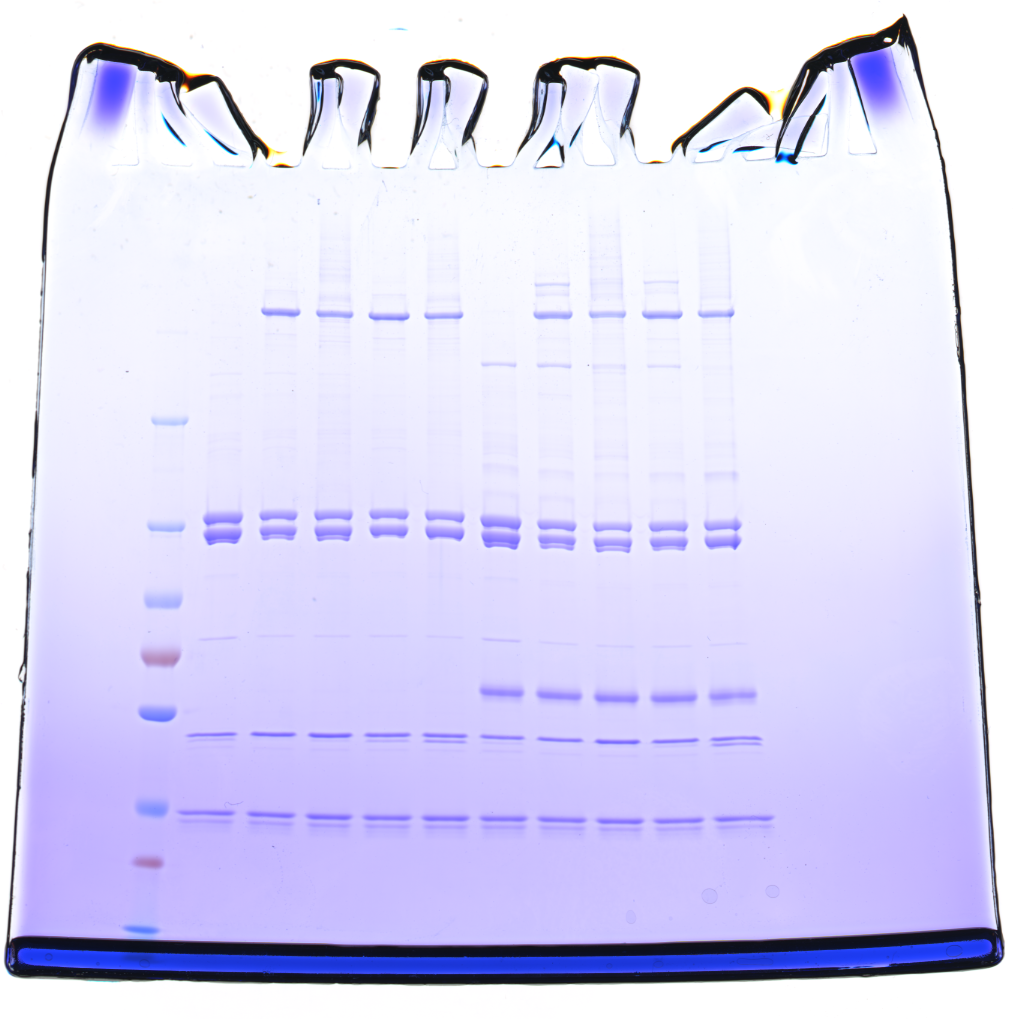

Supplement: Supplementary file 4 — Source Data for Expanded View [file EMBJ-40-e107807-s006.zip › Fig EV4B.tif]

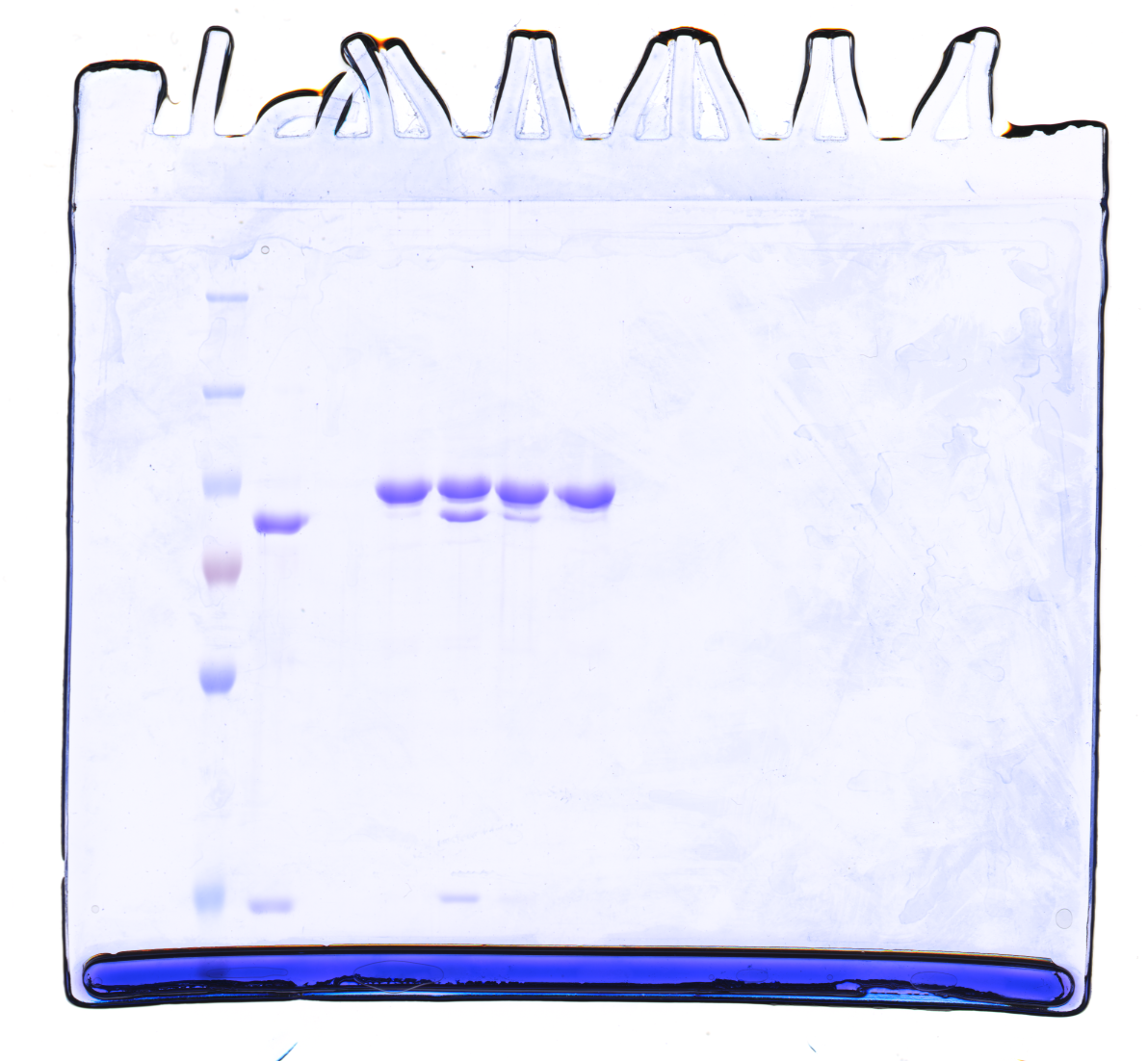

Supplement: Supplementary file 4 — Source Data for Expanded View [file EMBJ-40-e107807-s006.zip › Fig EV3F.tif]

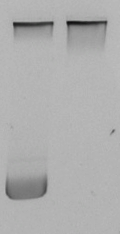

Supplement: Supplementary file 4 — Source Data for Expanded View [file EMBJ-40-e107807-s006.zip › Fig EV3E.png]

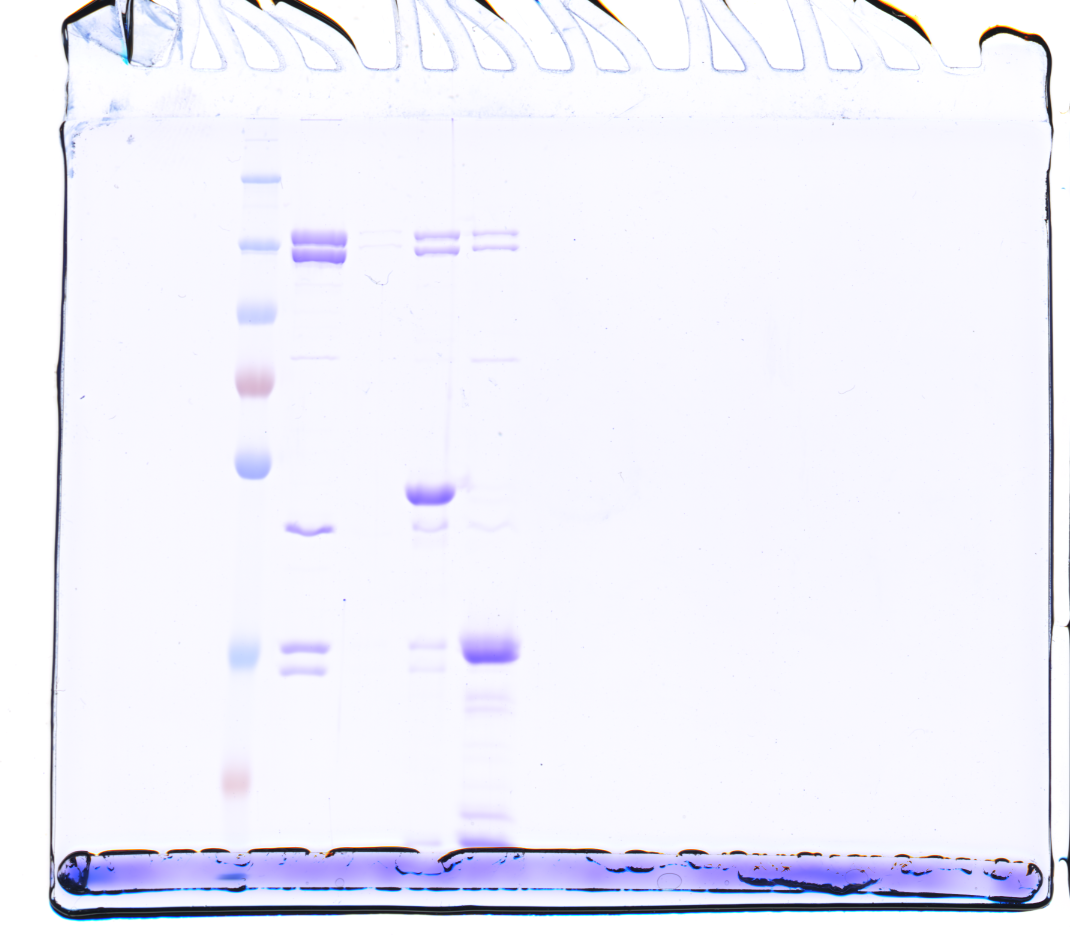

Supplement: Supplementary file 4 — Source Data for Expanded View [file EMBJ-40-e107807-s006.zip › Fig EV2C.tif]

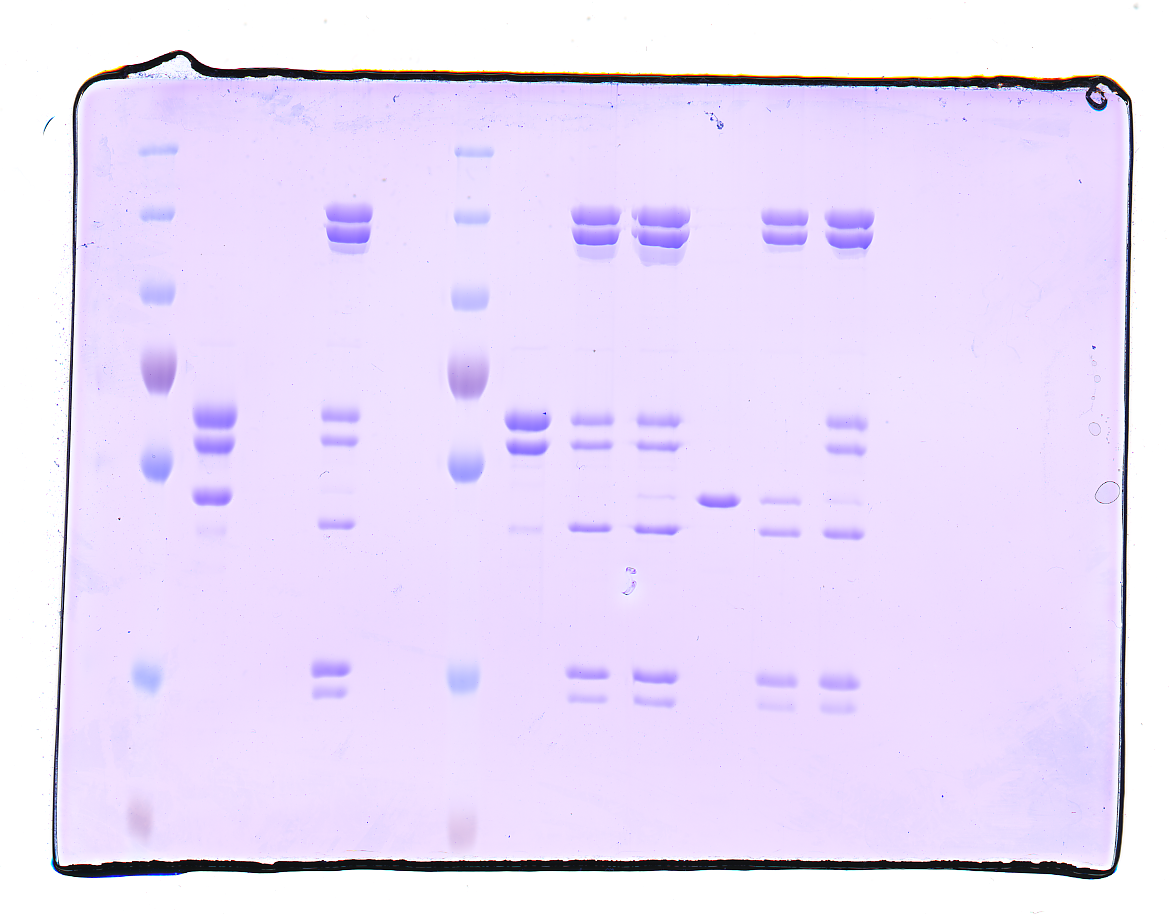

Supplement: Supplementary file 4 — Source Data for Expanded View [file EMBJ-40-e107807-s006.zip › Fig EV2A and B.tif]

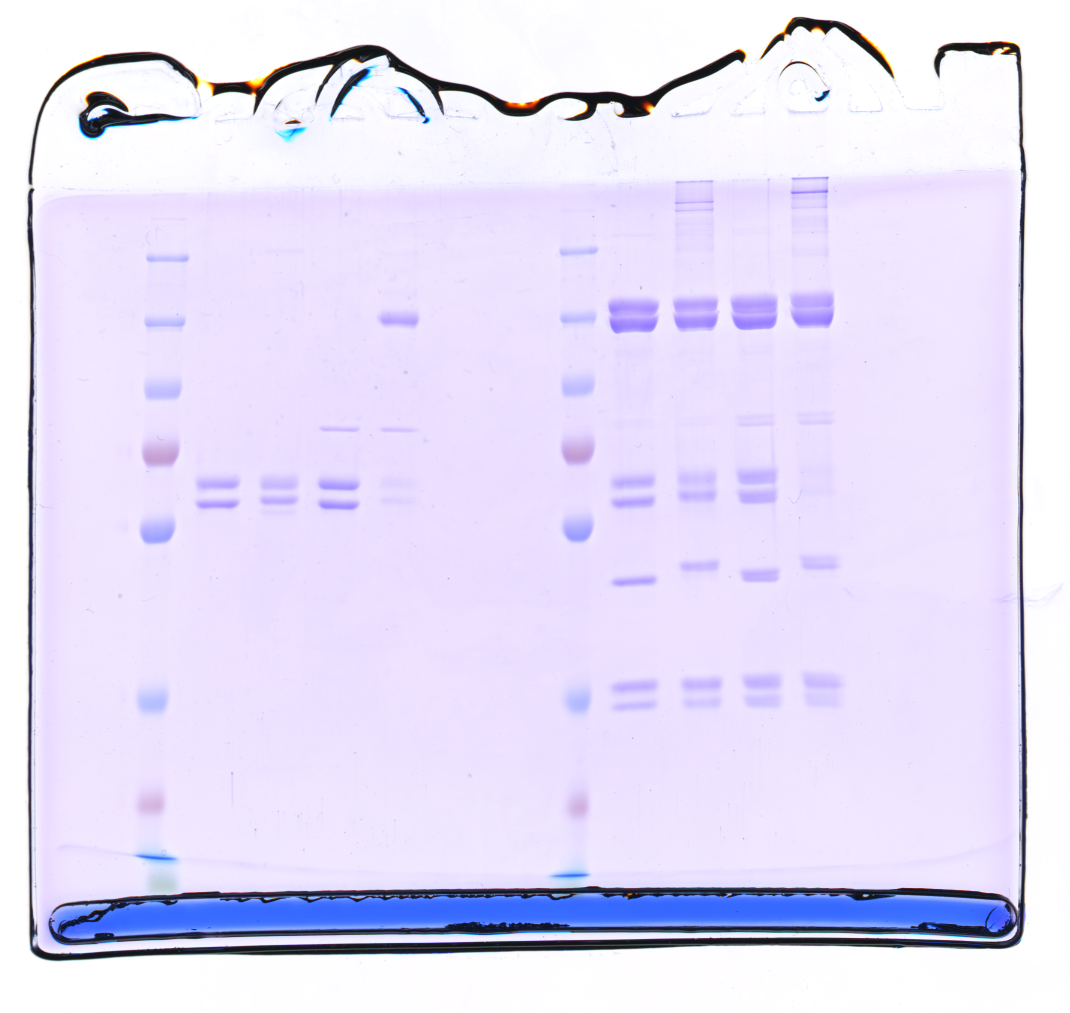

Supplement: Supplementary file 4 — Source Data for Expanded View [file EMBJ-40-e107807-s006.zip › Fig EV1D (left).tif]

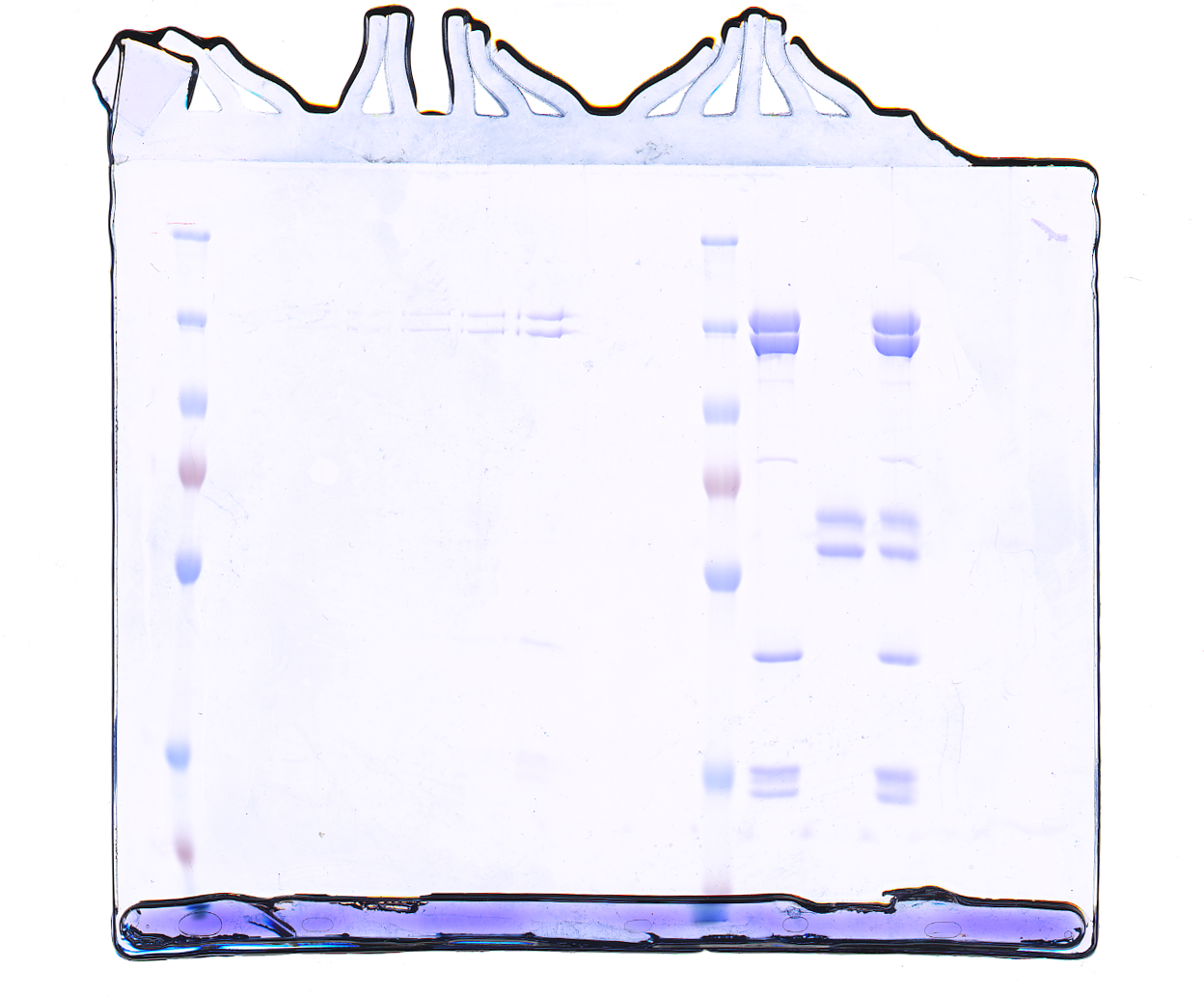

Supplement: Supplementary file 5 — Source Data for Figure 1A [file EMBJ-40-e107807-s001.tif]

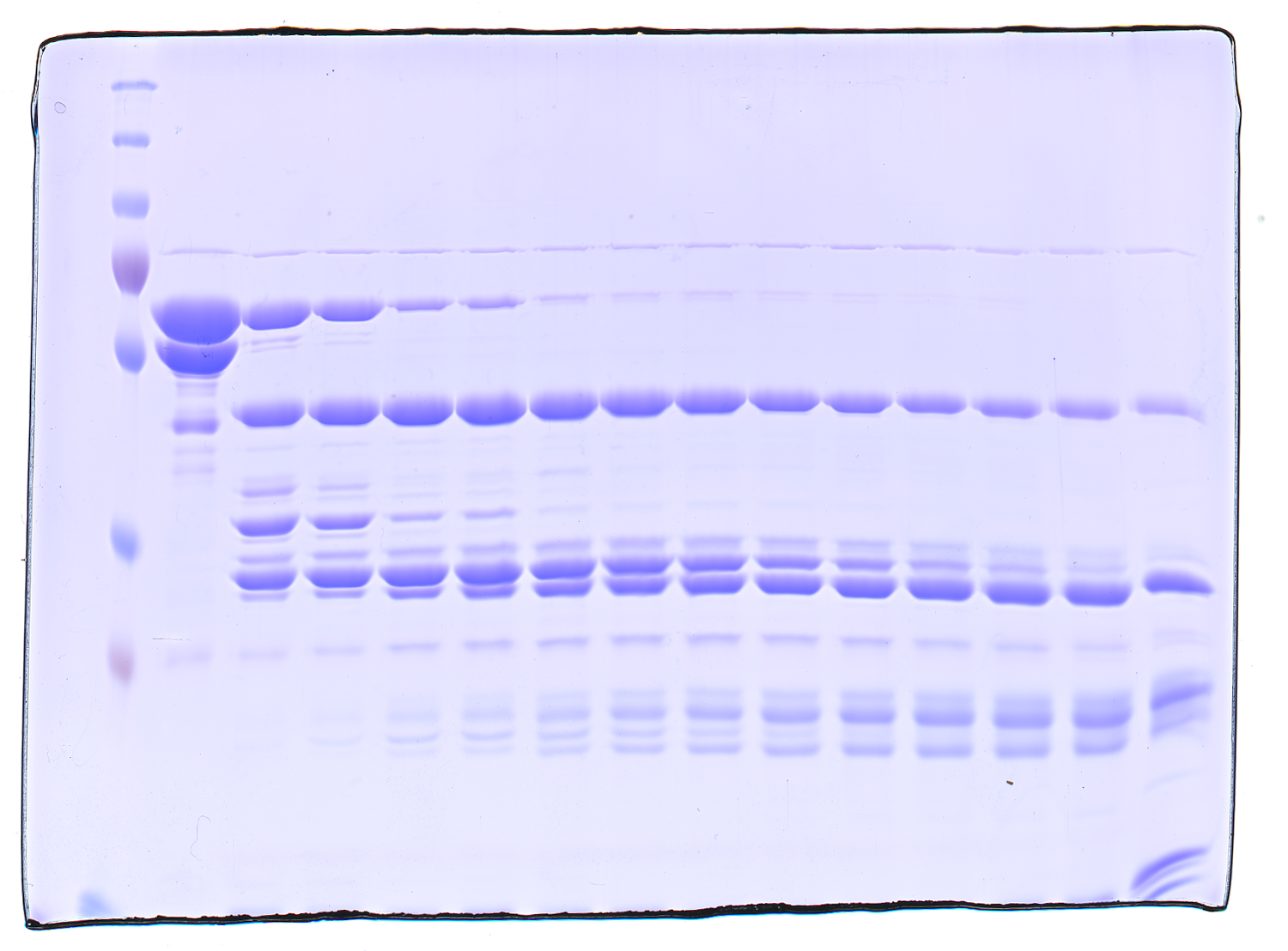

Supplement: Supplementary file 6 — Source Data for Figure 2B [file EMBJ-40-e107807-s004.tif]

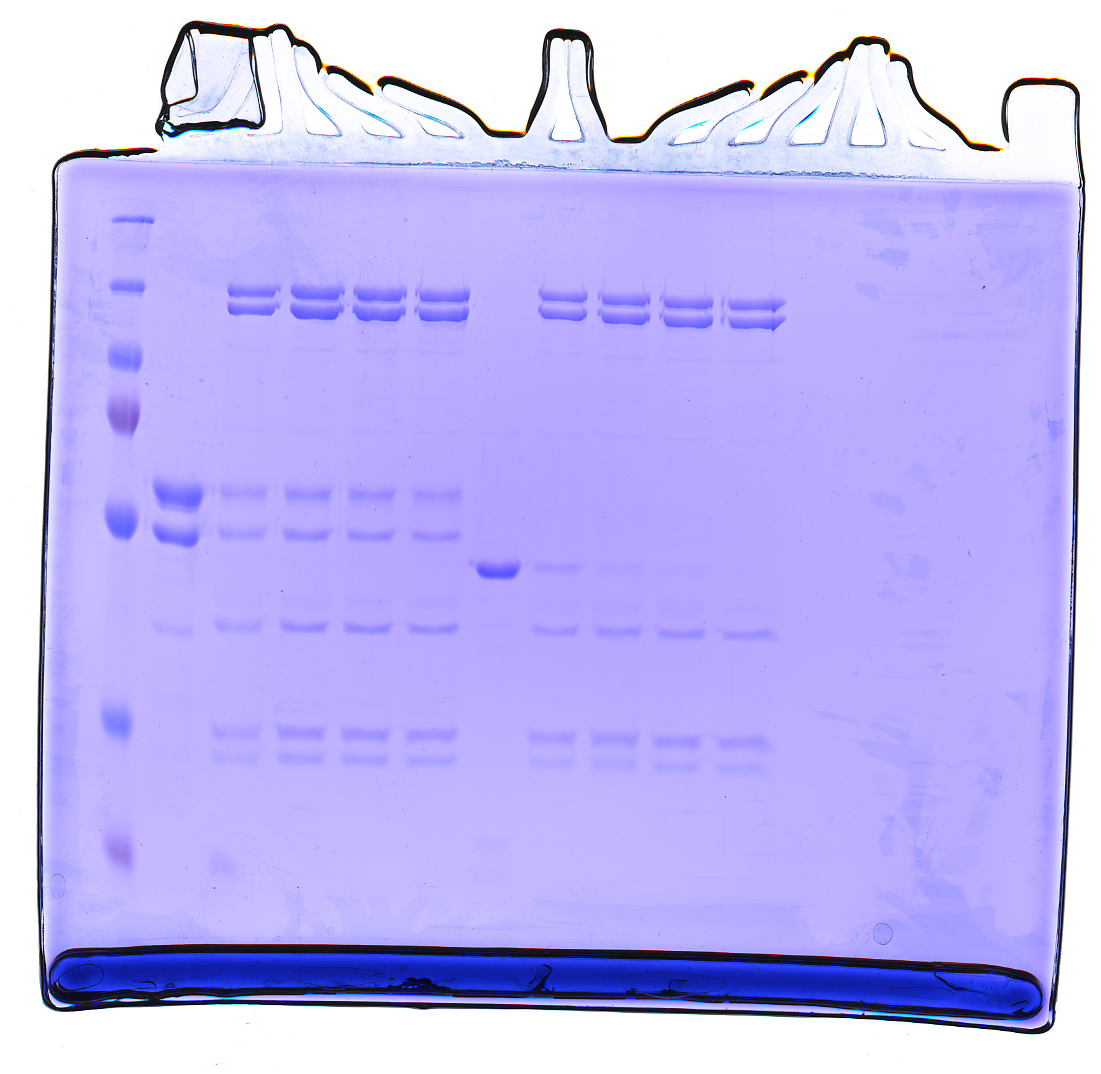

Supplement: Supplementary file 7 — Source Data for Figure 3 [file EMBJ-40-e107807-s007.zip › Fig 3B.tif]

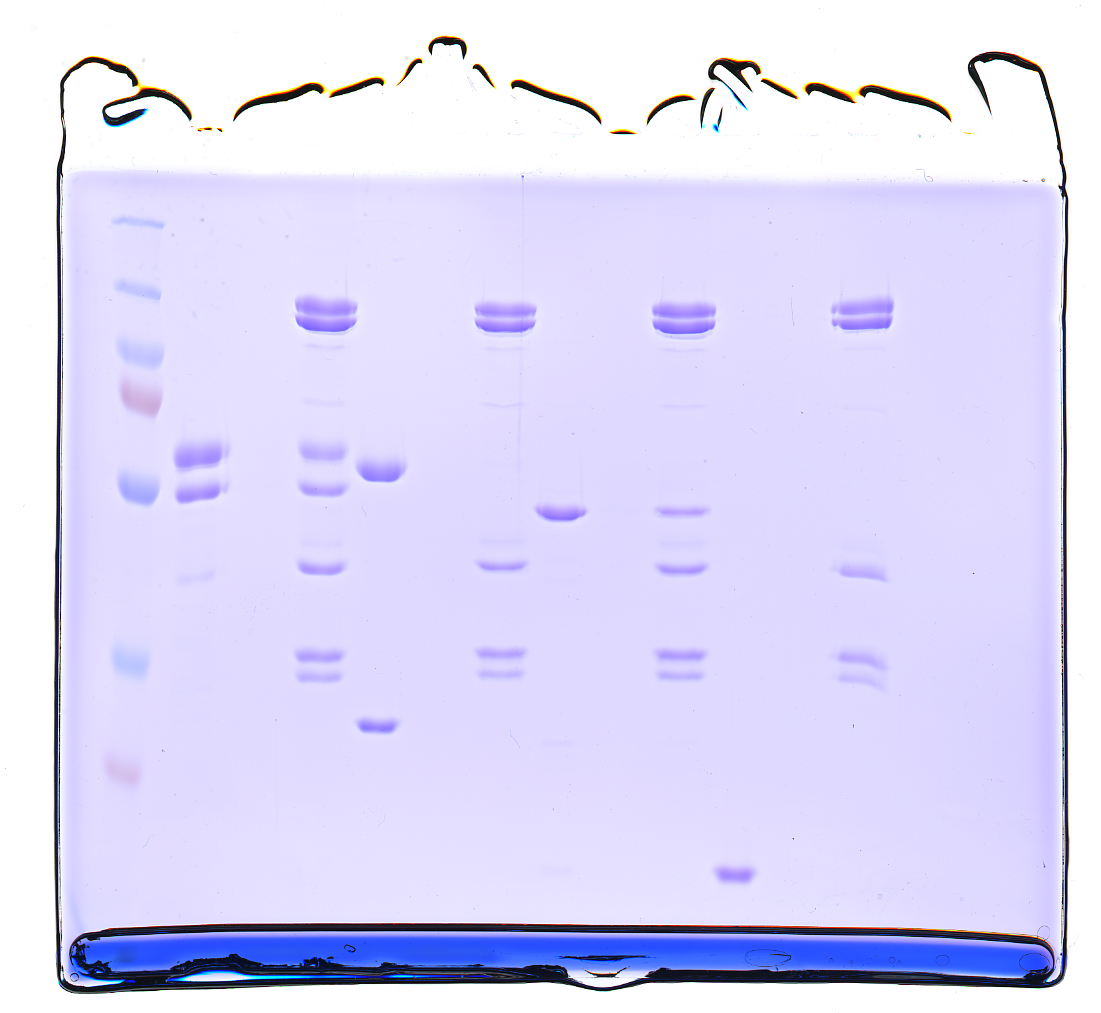

Supplement: Supplementary file 7 — Source Data for Figure 3 [file EMBJ-40-e107807-s007.zip › Fig 3A.tif]

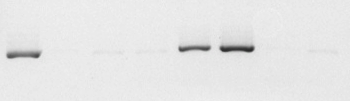

Supplement: Supplementary file 8 — Source Data for Figure 4 [file EMBJ-40-e107807-s005.zip › Fig 4F.TIF]

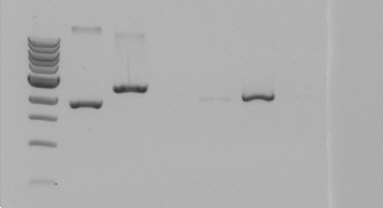

Supplement: Supplementary file 8 — Source Data for Figure 4 [file EMBJ-40-e107807-s005.zip › Fig 4E.TIF]

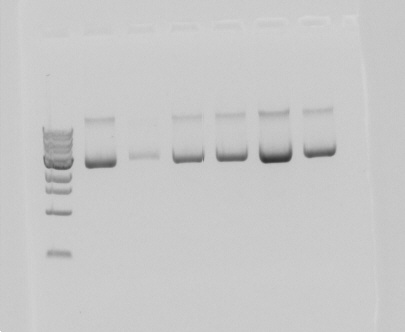

Supplement: Supplementary file 8 — Source Data for Figure 4 [file EMBJ-40-e107807-s005.zip › Fig 4D_DNA_lowsalt.TIF]

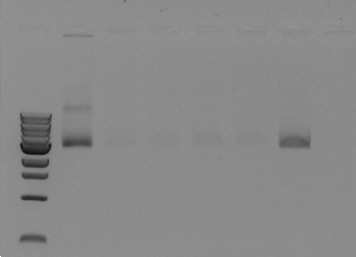

Supplement: Supplementary file 8 — Source Data for Figure 4 [file EMBJ-40-e107807-s005.zip › Fig 4D_DNA_highsalt.TIF]

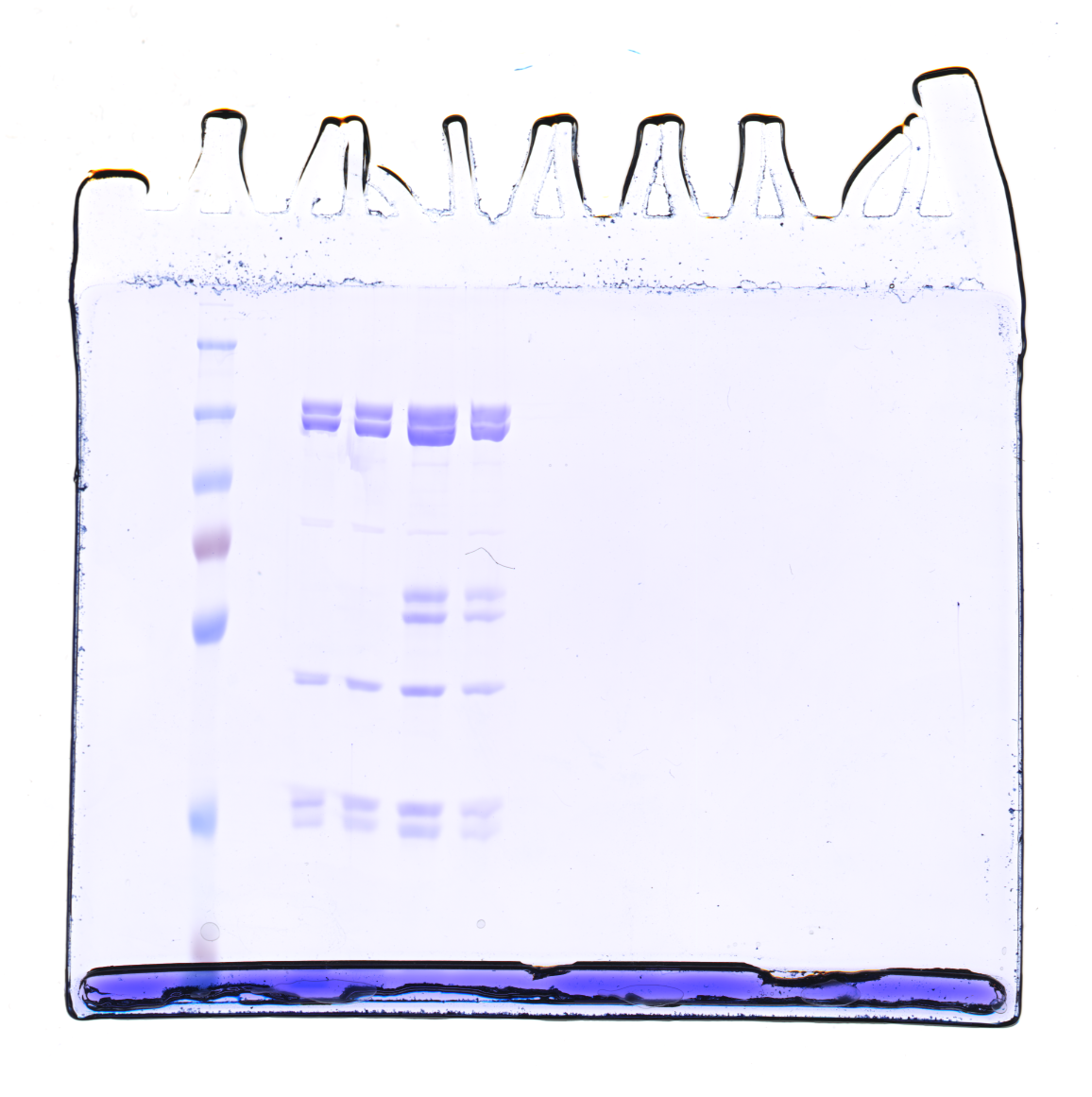

Supplement: Supplementary file 8 — Source Data for Figure 4 [file EMBJ-40-e107807-s005.zip › Fig 4D_Coomassie.tif]

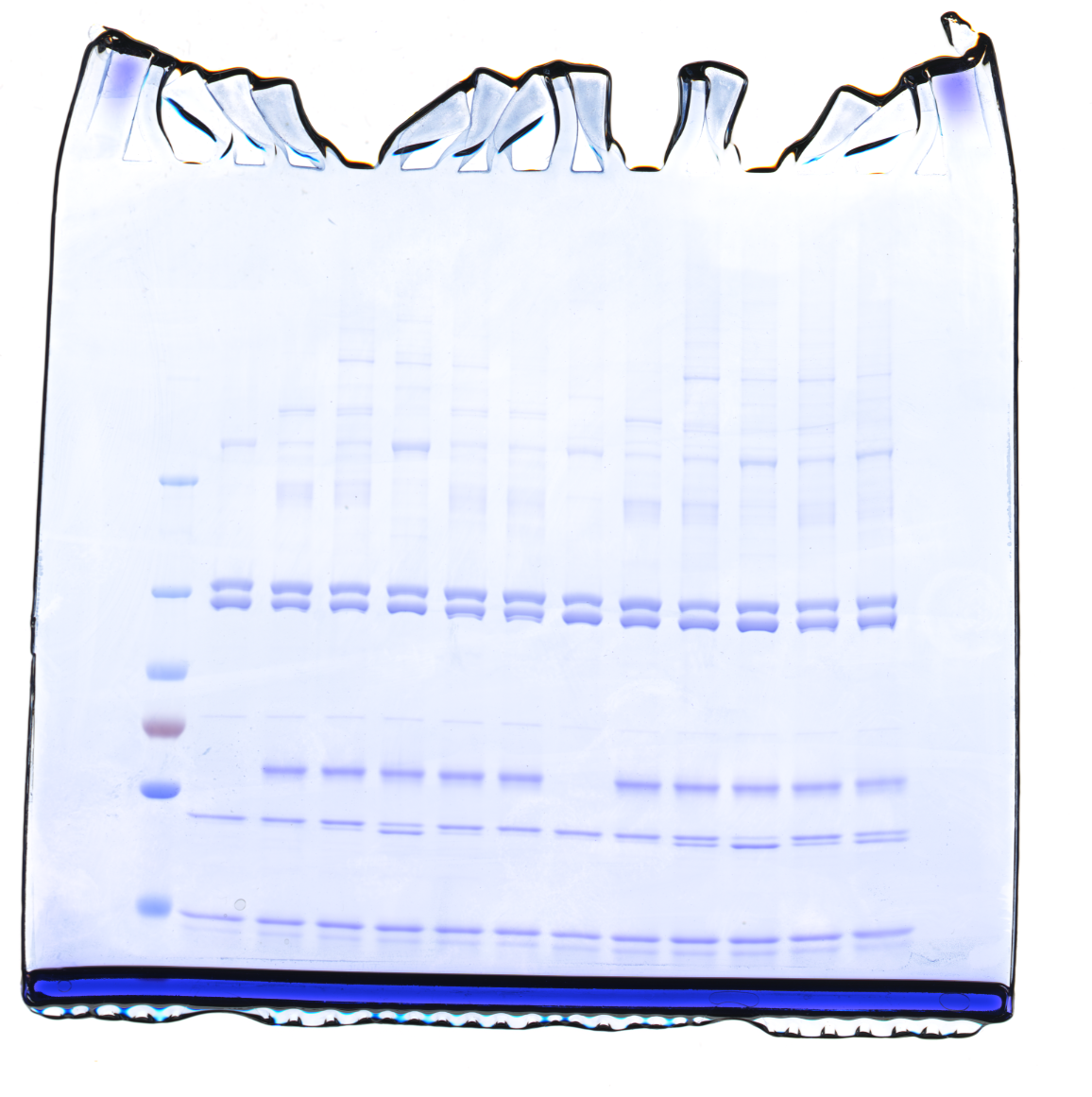

Supplement: Supplementary file 9 — Source Data for Figure 5 [file EMBJ-40-e107807-s009.zip › Fig 5F (left).tif]

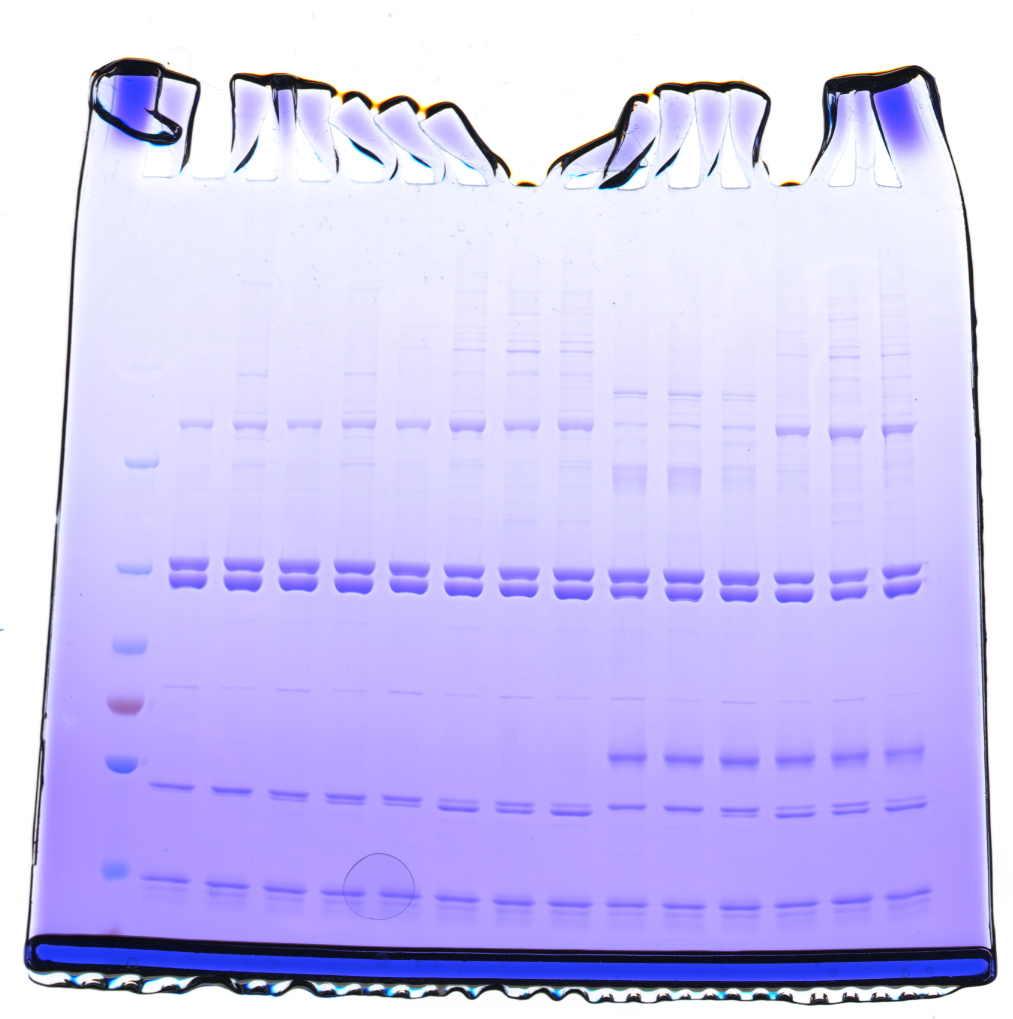

Supplement: Supplementary file 9 — Source Data for Figure 5 [file EMBJ-40-e107807-s009.zip › Fig 5E.tif]

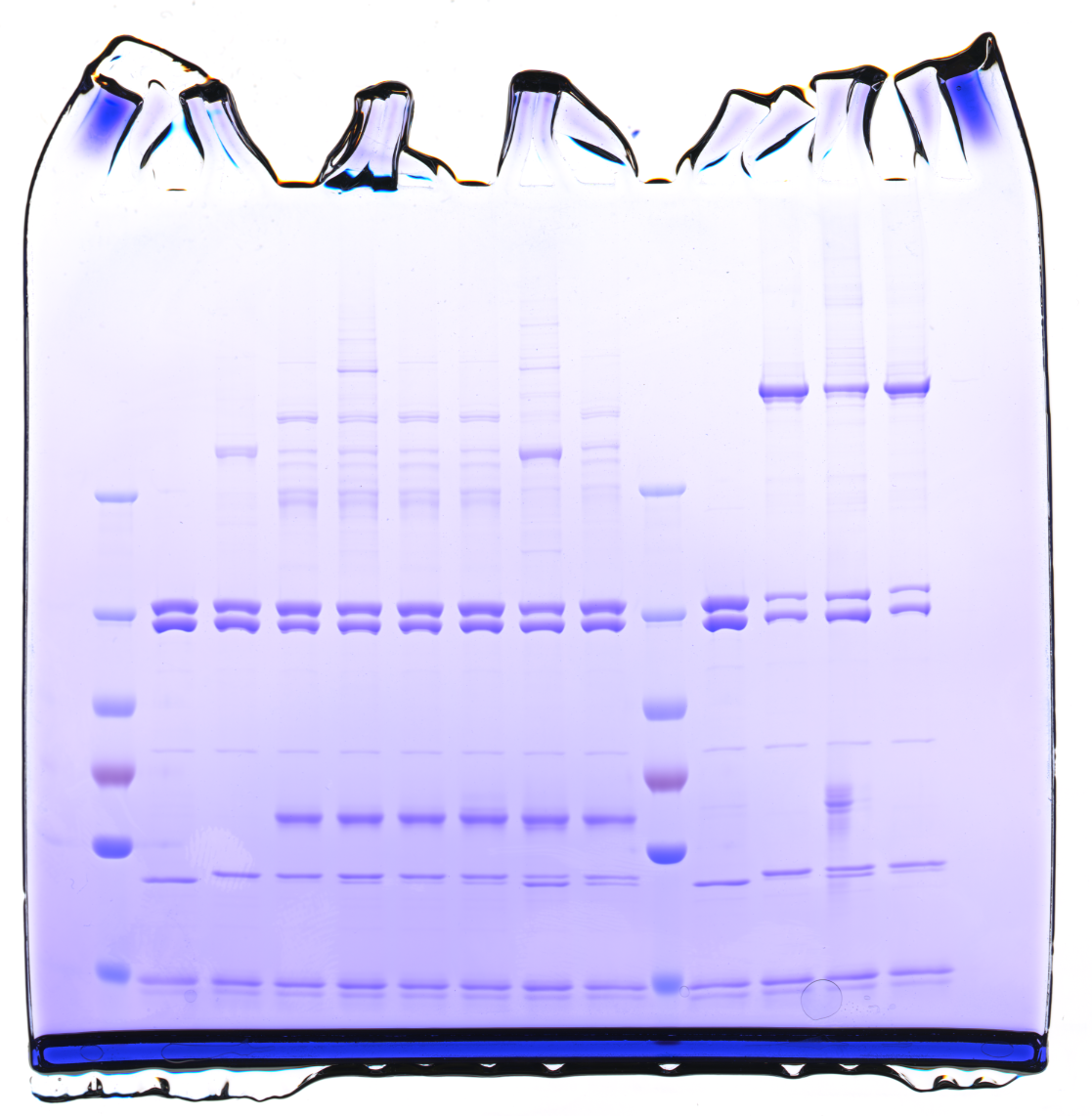

Supplement: Supplementary file 9 — Source Data for Figure 5 [file EMBJ-40-e107807-s009.zip › Fig 5C and D.tif]

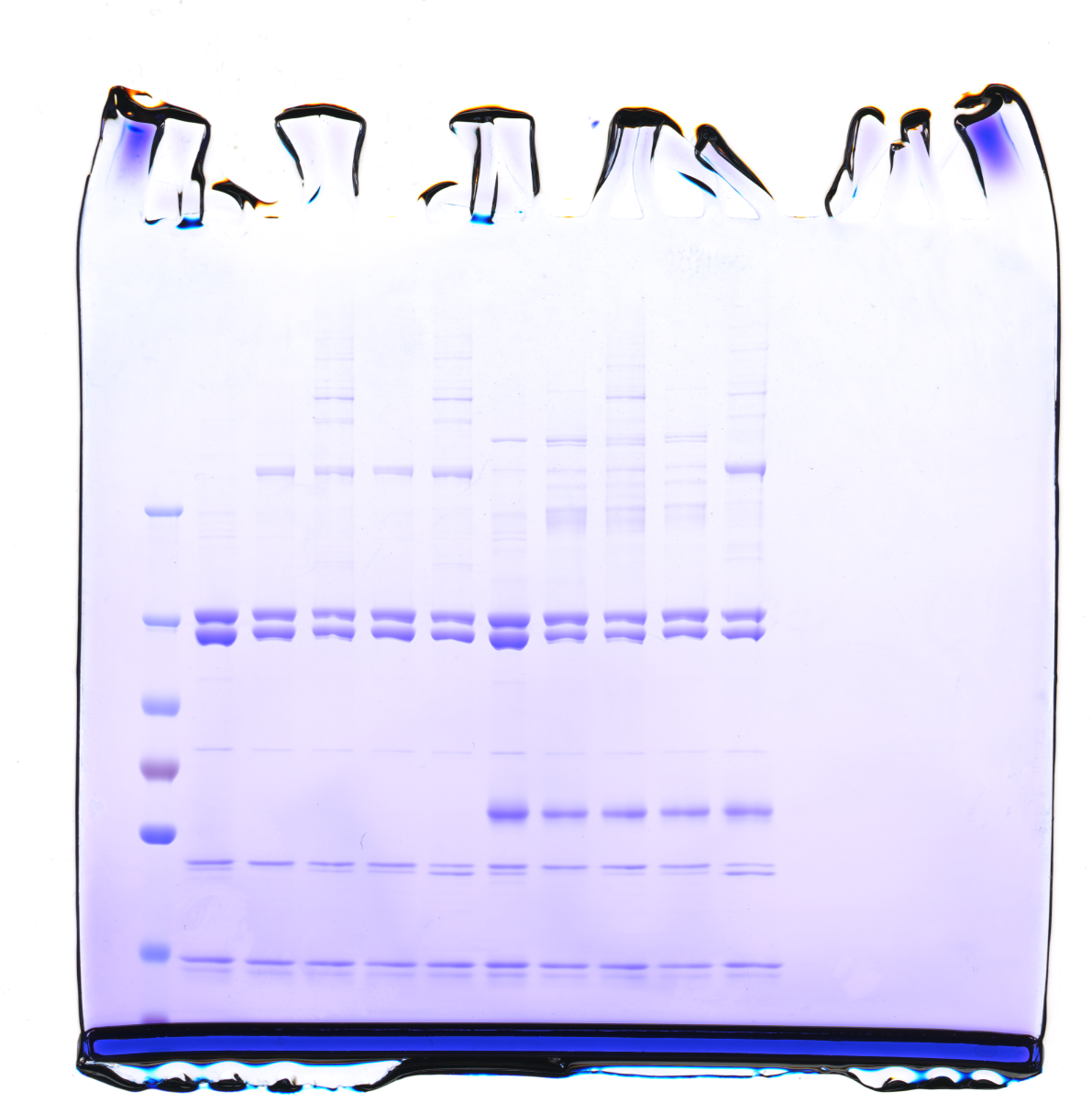

Supplement: Supplementary file 9 — Source Data for Figure 5 [file EMBJ-40-e107807-s009.zip › Fig 5B_4.tif]

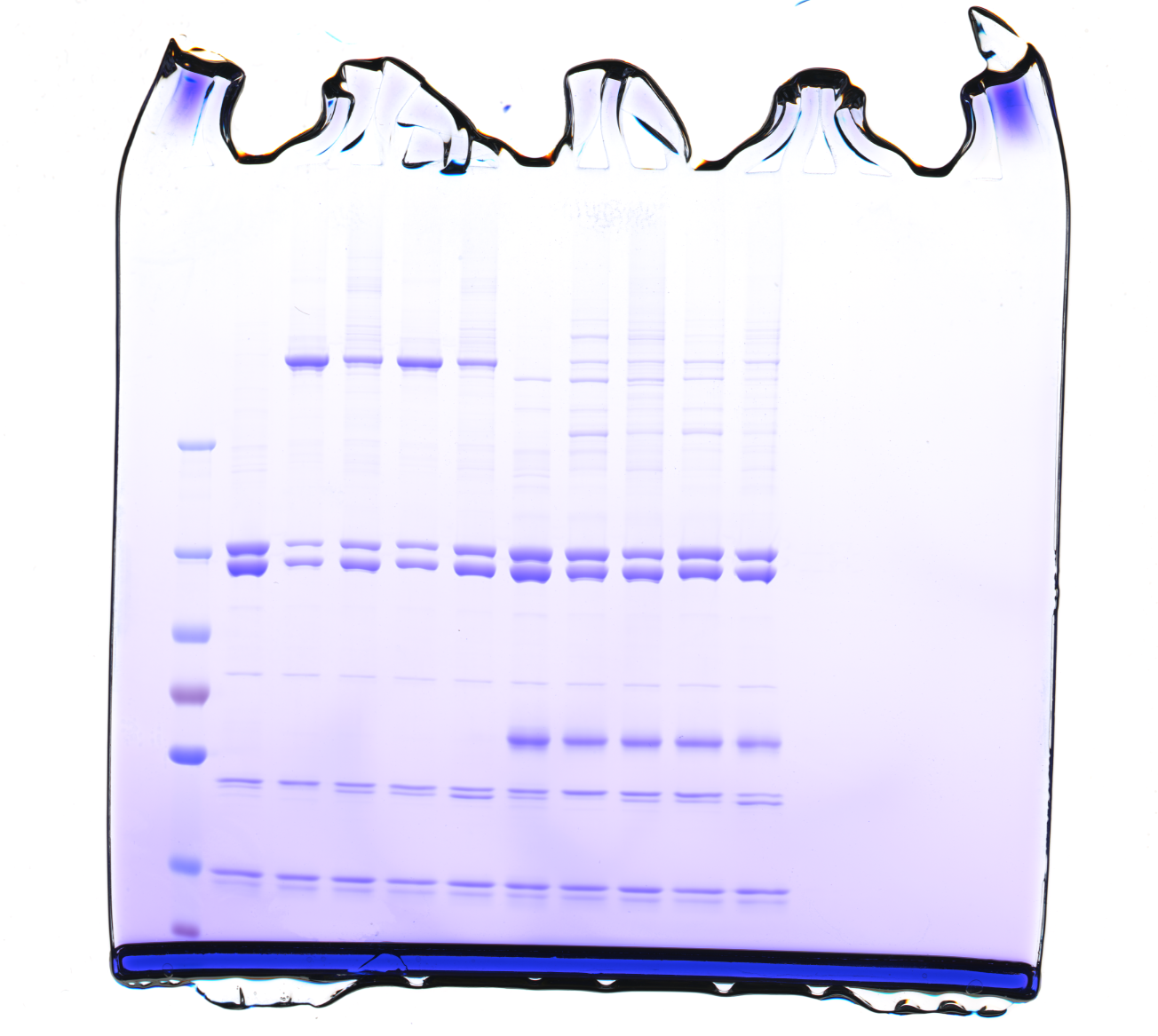

Supplement: Supplementary file 9 — Source Data for Figure 5 [file EMBJ-40-e107807-s009.zip › Fig 5B_3.tif]

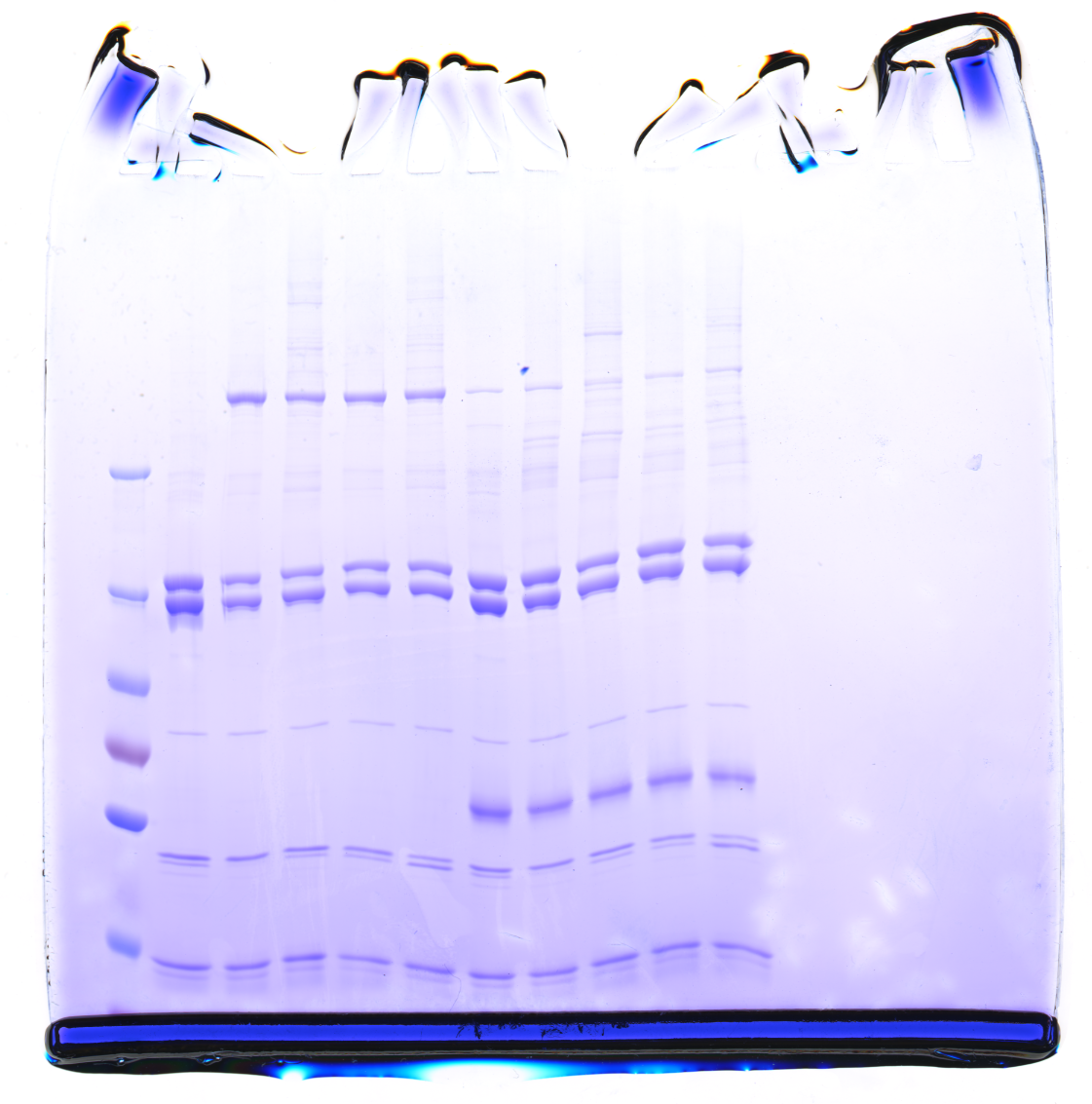

Supplement: Supplementary file 9 — Source Data for Figure 5 [file EMBJ-40-e107807-s009.zip › Fig 5B_2.tif]

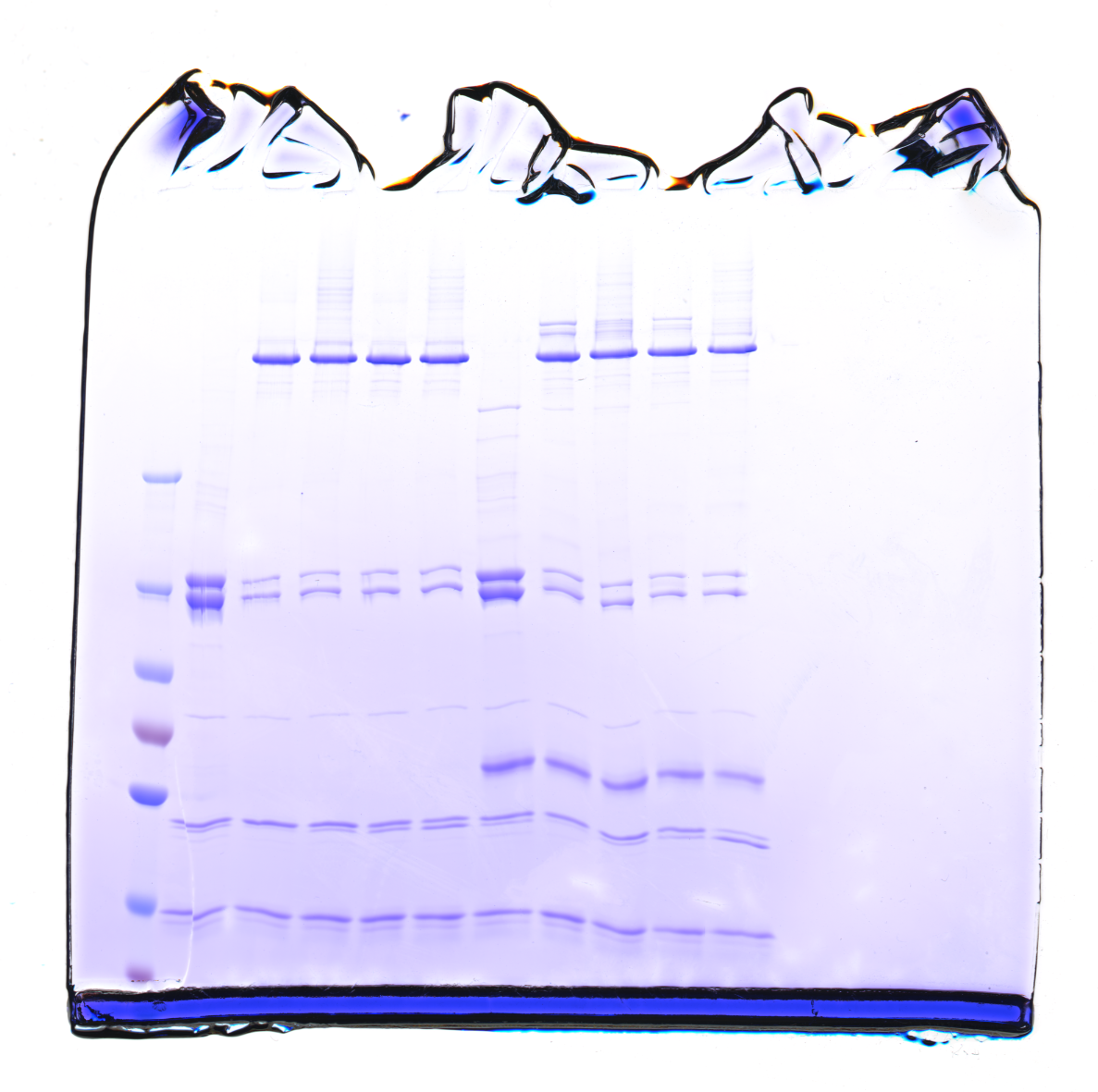

Supplement: Supplementary file 9 — Source Data for Figure 5 [file EMBJ-40-e107807-s009.zip › Fig 5B_1.tif]

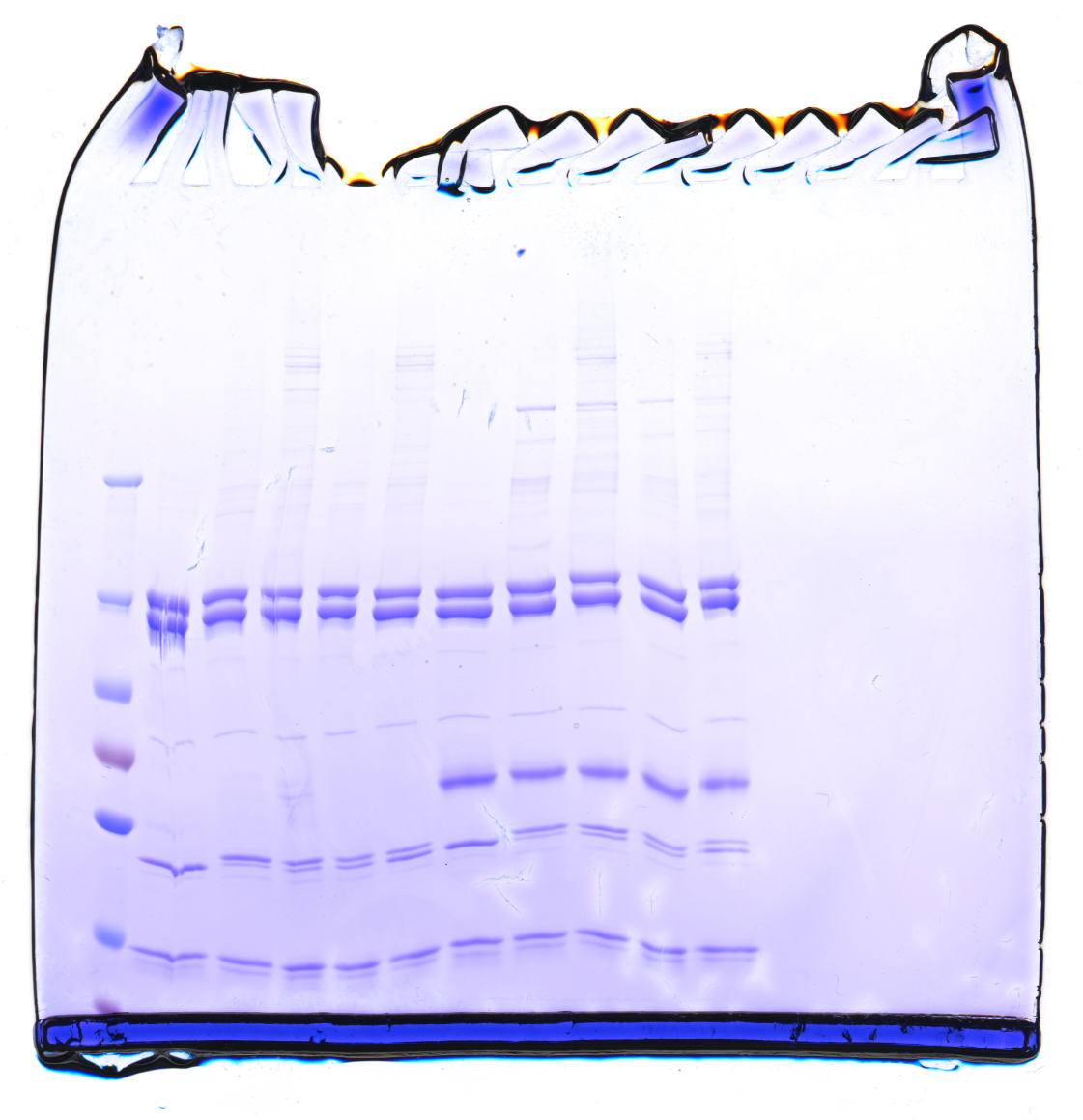

Supplement: Supplementary file 9 — Source Data for Figure 5 [file EMBJ-40-e107807-s009.zip › Fig 5A.tif]
